# The value of diffusion-weighted imaging in assessing the ADC changes of tissues adjacent to breast carcinoma

**DOI:** 10.1186/1471-2407-9-18

**Published:** 2009-01-14

**Authors:** Zhang Yili, Huang Xiaoyan, Du Hongwen, Zhang Yun, Chen Xin, Wang Peng, Guo Youmin

**Affiliations:** 1 Imaging center, the 1st Affiliated Hospital of Medical College, Xi'an Jiaotong University, Xi'an 710061, PR China; 2 Imaging center, the 2nd Affiliated Hospital of Medical College, Xi'an Jiaotong University, Xi'an 710004, PR China; 3 Department of Radiology of Beijing Chao-Yang Hospital, Captial Medical University, Beijing 100020, PR China

## Abstract

**Background:**

To define a threshold value of apparent diffusion coefficient (ADC) with which malignant breast lesions can be distinguished from benign lesions, and to evaluate the ADC change of peri-tumor tissue in breast carcinoma by echo planar-diffusion weighted imaging (EPI-DWI).

**Methods:**

57 breast lesions were scanned by routine MRI and EPI-DWI. The ADC values were compared between malignant and benign lesions. The sensitivity and specificity of EPI-DWI and the threshold ADC value were evaluated by Receiver Operating Characteristic curve (ROC). The ADC values of malignant lesion and layered peri-tumor tissues (from innermost layer 1 to outermost layer 4 with 5 mm every layer) in different directions were compared and the ADC values among different layers were compared.

**Results:**

The ADC value of 35 malignant lesions was statistically lower than that of 22 benign lesions (P < 0.05). In ROC curve, the threshold value was 1.24 +/- 0.25*10E-3 mm^2^/s (b = 500) or 1.20 +/- 0.25*10E-3 mm^2^/s (b = 1000). The ADC value of malignant lesions was statistically lower than that of peri-tumor tissues in different directions (P < 0.05). For peri-tumor tissues, the ADC values increased gradually from layer 1 to layer 4 and there was a significant difference between the ADC values of layer 1 and layer 2 (P < 0.05); while from layer 2 outwards, there was no statistical difference among different layers.

**Conclusion:**

ADC value was a sensitive and specific parameter that could help to differentiate benign and malignant breast lesions. ADC changes in tissues adjacent to breast carcinoma could be detected by EPI-DWI, which made EPI-DWI a promising method for helping to determine surgical scope of breast carcinoma.

## Background

Conservative surgery has become a well-established alternative to mastectomy in the treatment of breast cancer. However, in case of larger lesions or small-size breasts, the removal of adequate volumes of breast tissue to achieve tumor-free margins and reduce the risk of local relapse may compromise the cosmetic outcome, causing unpleasant results [1]. An issue of critical importance is thus to know well the transition from the tumor tissue to normal tissue in breast carcinoma in order to helpfully decide the surgical scope. Currently, the evaluation of tumor scope in clinical works relies on pathologic examination of margins free of gross tumor tissues. However, previous studies reported that in some patients with histologically negative margins, a relatively high recurrence rate was still observed [2]. It was thought that genetic and molecular alteration precedes phenotypic changes, therefore histologic assessment alone may be inadequate to detect the presence of transformed cells in surgical margins [3]. In another word, histologic assessment alone may be insufficient for the detection of transition from the tumor tissue to normal tissue. A recent study [4] discovered that there existed the geographic zones of the normal tissue adjacent to invasive cancers in which methylation changes could be identified and the authors summarized that multiple gene promoters could be used as a surrogate biomarker to define the molecular margin of lumpectomy in the future. Studies of other malignant lesions [3,5-9], including head and neck cancer, pancreatic cancer, lung cancer, colorectal carcinoma, rectal cancer and hepatocellular carcinoma, also demonstrated that there existed a molecular border of tumor tissues with various molecular methods. However, all of these studies were performed on the biopsy tissues or resected tissue specimens and no preoperative assessment of peri-tumor was reported. There is, therefore, a need for a non-invasive technique to detect the change of peri-tumor tissue for the purpose of providing more information about surgical scope.

Magnetic resonance imaging (MRI) has a high resolution and can provide much more detailed images than mammography and ultrasonography (US), which makes it a widely-used tool for the diagnosis of breast lesions. However, the conventional breast MRI (plain MRI) is still a morphological diagnostic technique which only provides general anatomical information such as signal, shape, size and location. One of the latest advancements in MRI technology is the application of diffusion-weighted imaging (DWI). The principle underlying DWI is that the thermal motion of water molecules in extracellular fluid enables the acquisition of images that reflect both histological structure and cellularity [10] and therefore it can detect the changes of tissue structure at molecular level. It also enables the quantitative evaluation of apparent diffusion coefficient (ADC), which may be useful for distinguishing malignant from benign tissues and monitoring therapeutic outcome [11,12]. Compared with benign lesions, diffusion of malignant tumors with high cellular tissue decreased and the ADC value in malignant tumors is lower than that of benign lesions [13-15]. Several latest studies have shown that ADC has a potential for clinical appreciation in differentiating benign and malignant lesions with good specificity [16-19].

On the other side, DWI, based on its imaging mechanism, could detect the changes of ADC in different tissues. Measurement of the ADC provides a quantitative estimate of the restrictive nature of the motion of water molecules within tissue for each voxel in a diffusion-weighted image. This study was thus designed to compare the ADC values between malignant and benign lesions in breast and to study the change of ADC values in peri-tumor tissues through echo planar diffusion weighted imaging (EPI-DWI). Our main aim was to study the DWI changes in tissues adjacent to breast carcinoma, which would be helpful for the clinical surgeon to decide the scope and pattern of operation.

## Methods

This study was approved by the ethics committee of Xi'an Jiaotong University and all patients gave written informed consent before beginning the study.

### Patients

54 cases with 57 breast lesions were examined by ultrasound and/or mammography before MRI from June 2006 to January 2007 in our hospital. No previous chemotherapy or radiotherapy or surgery was administrated to these patients. All patients were female and aged between 31 and 77 years (mean age: 46.3 years). All patients underwent surgical resection and received definite pathological diagnosis in our hospital.

### Histological details

Findings were 22 benign lesions in 21 cases, including 14 breast fibroadenoma and 8 breast hyperplasia. Malignant lesions totalled 35 in 33 patients, including 31 infiltrating ductal carcinoma (IDC), 3 ductal carcinoma in situ (DCIS) and 1 breast malignant phyllode. Lymph nodes metastasis in malignant lesions was observed in 13 cases. The mean of largest diameter of the benign lesions was 38.0 mm (from 8 to 106 mm) and that of breast cancer was 28.4 mm (from 10 to 55 mm) and the largest diameter of 1 breast malignant phyllode was 72.4 mm.

### MRI protocols and imaging

**Figure 1:**
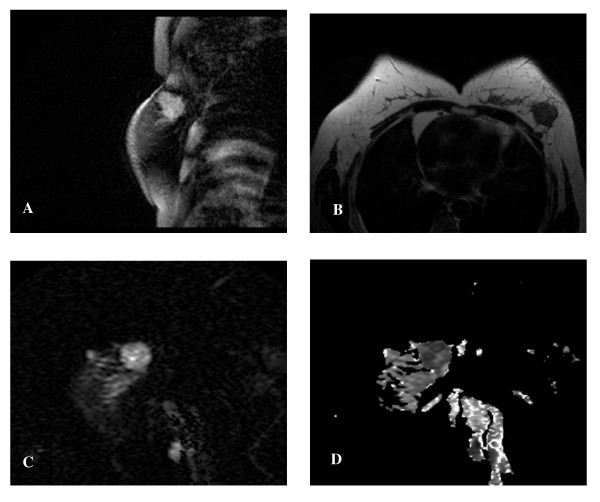
**45-year-old woman with IDC in the left breast**. A: WATS T1-weighted sagittal scan showed irregularly mass in the left breast with hyperintense signal. B: T2-weighted transverse scan showed the lesion was hypointense with spicule sign. C: EPI-DWI sagittal scan showed lesion with slightly inhomogeneity hyperintense in DWI. D: Inhomogeneity hypointense was found in ADC map (b = 500) and the ADC was 0.94 ± 0.19 ± 10^-3^mm^2^/s.

**Figure 2:**
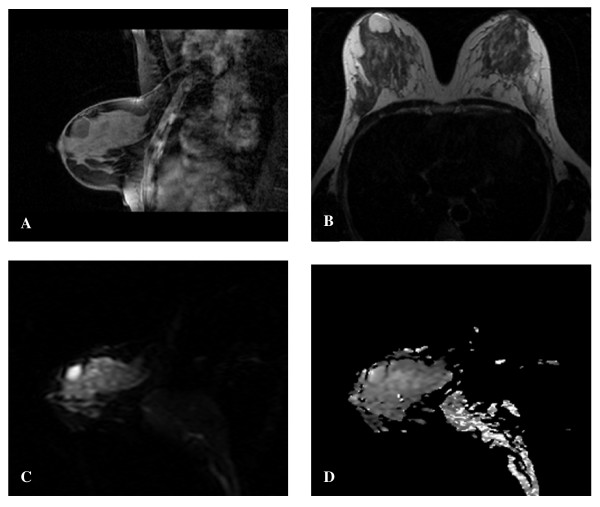
**36-year-old woman with fibroadenoma in right breast**. A: WATS T1-weighted sagittal scan showed a regular round slightly hypointense lesion. B: T2-weighted transverse scan showed a round and smooth hyperintense lesion in the right breast. C: EPI-DWI sagittal scan showed a hyperintense lesion in DWI. D: The isointense lesion was in ADC map (b = 1000) and the ADC was 2.44 ± 0.362×10^-3^mm^2^/s.

MRI was performed with a 1.5 T MR system (Philips, Gyroscan NT Release6, Netherlands) and a dedicated phased-array bilateral breast coil, with the patient lying prone and the breast in a holder to reduce motion. The imaging protocols included a sagittal T1-weighted (T1-W) water selective excitation (WATS) first field echo (FFE) pulse sequence (matrix = 512^2^, slice thickness = 5 mm, Flip angle(deg) = 25.00); a T2-weighted transverse (T2-W) turbo spin-echo (TSE) pulse sequence (matrix = 512^2^, slice thickness = 5 mm, Flip angle(deg) = 90.00); and an Gradient echo planar image (EPI) DWI pulse sequence (matrix = 128, FOV(mm) = 350.00, slice thickness = 5 mm, Flip angle(deg) = 90.00, diffusion mode = SE, NSA = 1). We modified b-values (b = 500 second/mm^2 ^and 1000 second/mm^2^) to assume a better-fit convergence. Finally we obtained two trace-weighted images and ADC maps reconstruction. The analysis of magnetic resonance images of the 57 lesions was shown in Table 1 (Figure 1, 2).

**Table 1:** Analysis of magnetic resonance images of the 57 lesions

Classification	No.	WATS T1-weighted	T2-weighted SE	EPI-DWI	ADC map
Malignant lesions	35				

*Breast cancer*	33	Slightly hyperintense	Hypointense	Slightly inhomogeneity hyperintense	Inhomogeneity hypointense
	
	1	Isointense	Isointense	Hyperintense	Hyperintense

*Breast sarcoma*	1	Slightly hypointense	Inhomogeneity hyperintense	Slightly inhomogeneity hyperintense	Slightly inhomogeneity isointense

Benign lesion	22				

*Fibroadenoma*	3	Homogeneity hypointense	Homogeneity hyperintense	Homogeneity hyperintense	Homogeneity hyperintense
	
	8	Homogeneity isointense	Hypointense (2) Isointense (4) Hyperintense (2)	Hyperintense (7) Isointense (1)	Isointense

	3	Homogeneity hyperintense	Hypointense	Hyperintense (2) Hypointense (1)	Isointense (2) Inhomogeneity Hyperintense (1)

*Breast hyperplasia*	8	Densification of gland (6) Hypointense (1) Isointense (1)	Hyperintense (2)	Hyperintense (2)	Hyperintense (1) Hypointense (1)

### ADC value

All ADC values were calculated according to the formula: ADC = -(1/b)In(S/So), where So and S are the signal intensities in the region of interest (ROI), obtained with different gradient factors (b values of 500, and 1000 second/mm^2^). ADC distribution was demonstrated on an ADC map created with Easy Vision Workstation (Philips, Netherlands). The ROI was placed in and around the target lesion and the size of ROI in the lesion was 5 mm in diameter. The protocol in measuring ADC values by two readers was technologically same.

### Statistical analysis

The Independent-Sample *T*-test and One-Way ANOVA were used to determine statistical significance. *P *value < 0.05 was regarded as statistically significant. All above statistical analyses were performed using the SPSS11.5 software package (SPSS Inc, Chicago, IL, USA). Cut-off values between benign and malignant lesions were defined using Receiver Operating Characteristic (ROC) curves.

## Results

### Comparison of ADC values between malignant lesions and benign lesions

Either b = 500 or b = 1000, the ADC values from reader 1 and reader 2 were highly consistent in all breast lesions: for malignant lesions, when b = 500, *P *= 0.985 and when b = 1000, *P *= 0.890; while for benign lesions, when b = 500, *P *= 0.839, and when b = 1000, *P *= 0.764. (all *P *> 0.05) (Table 2). Furthermore, there was no statistical difference between b = 500 and b = 1000 in malignant lesions or benign lesions (both *P *> 0.05).

**Table 2:** Average ADC values (×10^-3^mm^2^/s) measured by reader 1 and reader 2

	Reader1		Reader2	
	
	b = 500	b = 1000	b = 500	b = 1000
Malignant lesions	1.04 ± 0.24^a^	1.01 ± 0.19^b^	1.04 ± 0.21	1.01 ± 0.22

Benign lesions	1.78 ± 0.31^c^	1.72 ± 0.36^d^	1.79 ± 0.28	1.75 ± 0.33

^a^*P *= 0.985, *vs *reader 2; ^b^*P *= 0.890, *vs *reader 2; ^c^*P *= 0.839, *vs *reader 2; ^d^*P *= 0.764, *vs *reader 2.

**Figure 3:**
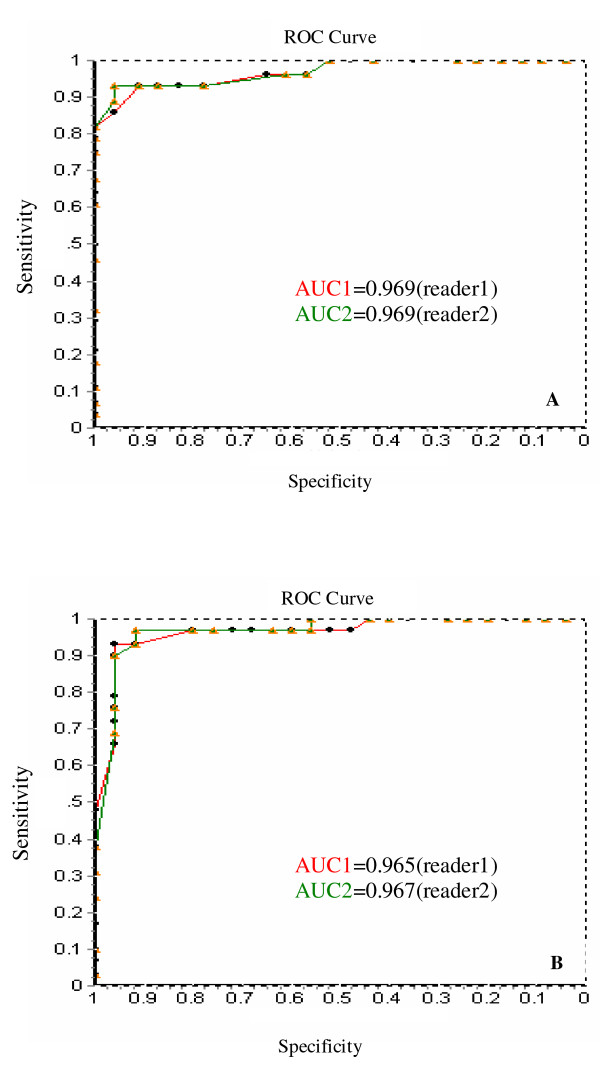
**A: ROC curves for the ADC values from reader1 and reader2 (b = 500). The area under the ROC curve (AUC) is 0.969(reader1) and 0.969(reader2)**. B: ROC curves for the ADC values from reader1 and reader2 (b = 1000). The area under the ROC curve (AUC) is 0.965 (reader1) and 0.967 (reader2).

All data were entered into a spreadsheet and ROC methodology was then used to decide the ADC value threshold which could differentiate malignant lesions from benign lesions. ROC curves from both readers were represented in Figure. 3A and 3B. The threshold values of ADC were 1.24 ± 0.25 ± 10^-3^mm^2^/s (b = 500) and 1.20 ± 0.25 ± 10^-3^mm^2^/s (b = 1000), respectively, and the summary of ROC curves about their corresponding sensitivity and specificity were presented in table 3.

**Table 3:** The summary of ROC curve

	Reader1		Reader2		Average of both readers	
	
	b = 500	b = 1000	b = 500	b = 1000	b = 500	b = 1000
Threshold(×10^-3^mm^2^/s)	1.29 ± 0.25	1.22 ± 0.25	1.39 ± 0.25	1.38 ± 0.25	1.24 ± 0.25	1.20 ± 0.25

Sensitivity	93%	93%	93%	97%	93%	96%

Specificity	91%	96%	96%	92%	100%	97%

However, the ADC values of malignant lesions and benign lesions were statistically different (both *P *< 0.001 for b = 500 and b = 1000, Table 4), which is obviously higher in benign lesions than that in breast malignant lesions, except for one malignant phyllode, whose ADC value was very close to that of benign lesions.

**Table 4:** Values of ADC measured in breast lesions

Histology	No.	ADC(range) × 10^-3^mm^2^/s b = 500	ADC(range) × 10^-3^mm^2^/s b = 1000
Malignant lesions	35	1.04 ± 0.23^a^	1.01 ± 0.20^b^

*ductal carcinoma in situ*	3	0.99 ± 0.18	0.97 ± 0.21

*infiltrating ductal carcinoma*	31	1.04 ± 0.26	1.02 ± 0.19

*Sarcoma*	1	1.72 ± 0.21	1.72 ± 0.13

Benign lesions	22	1.79 ± 0.29	1.73 ± 0.34

^a^*P *= 0.000, *vs *benign lesions; ^b^*P *= 0.000, *vs *benign lesions.

### ADC values among different layers and different directions around malignant lesions

**Figure 4:**
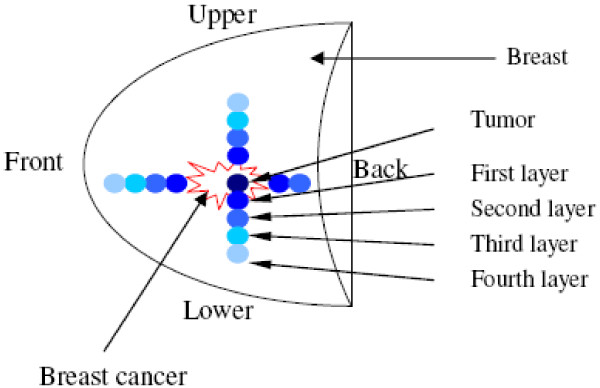
**This sagittal schematic drawing showed how we measured the ADC values of tumor tissue and peri-tumor tissues**. The red crooked line presented the breast tumor and the small circles of different colors from dark blue to light blue presented different layers of peri-tumor tissues with a diameter of 5 mm.

Both readers further evaluated the ADC values of different layers in different directions around the lesions (Figure 4). Since the ADC map in our study was based on a sagittal imaging, only one ADC map with the biggest diameter of tumor was selected and the center of tumor was set as the point to define the upper and lower in vertical direction as well as anterior and posterior in horizontal direction and the ROI distribution was symmetric in opposite directions. Since slice thickness of all above sequences was 5 mm, from the anatomic border of malignant tumor outwards, ADC value of each ROI layer with a thickness of 5 mm was measured till skin or until the ADC values of adjacent layers were very close to each other. The outermost layer in this study was layer 4.

**Figure 5:**
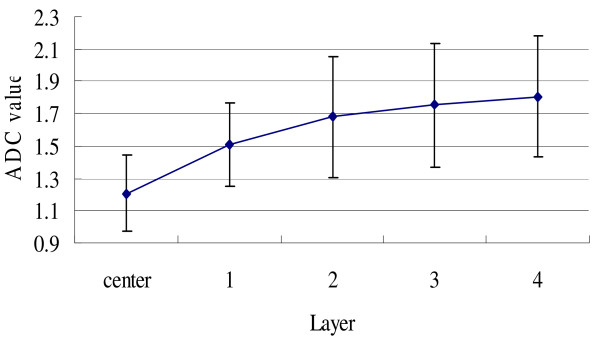
**The ADC values (×10^-3^mm^2^/s) of tumor tissue and peri-tumor tissues in different directions**. The standard deviation (×10^-3^mm^2^/s) was as following: front: 0.418, back: 0.509, upper: 0.289, blow: 0.374, central: 0.155.

Our results showed that the ADC of malignant lesions was statistically lower than that of peri-tumor tissues (from layer 1 to layer 4) in all directions (Figure 5) (*P *< 0.05), while there was no statistical difference in ADC values among the four directions within same layer.

**Figure 6:**
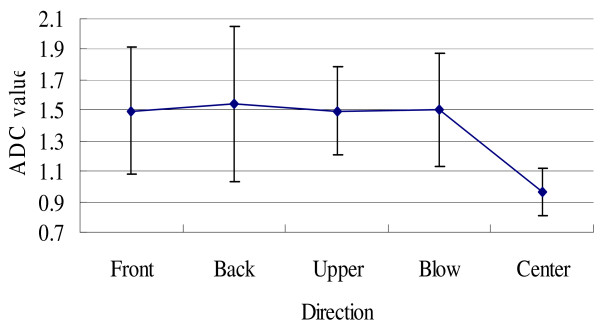
**The ADC values (×10^-3^mm^2^/s) of tumor tissue and peri-tumor tissues of different layers**. The standard deviation (×10^-3^mm^2^/s) was as following: tumor: 0.233, first layer: 0.257, second layer: 0.370, third layer: 0.382, fourth layer: 0.370.

Further detailed analysis showed that from layer 1 to layer 4, the ADC value increased gradually (Figure 6). The ADC value of tumor lesion was significantly lower than that of each layer of peri-tumor tissues, while the ADC value of layer 1 was significantly lower than that of layer 2 (*P *< 0.05). However, there was no significant difference between the ADC values of layer 2 and layer 3, or layer 3 and layer 4. Moreover, we also compared the ADC values of different layers with the normal contralateral breast tissue. We found that only ADC value of layer 1 was significantly lower than that of contralateral breast tissue (*P *< 0.05).

## Discussion

Despite the improvement in the detection of breast cancer with the widespread application of mammography and ultrasound, breast lesions still remain difficult to diagnose and characterize, especially in dense fibroglandular breasts. The main advantage of MRI in the breast is that they can improve the detection and characterization of multiple and/or small lesions even in the dense fibroglandular breasts. However, the low specificity of MRI remains a problem [20].

In recent years, the DWI has been extensively applied in evaluating cerebral tumors and the correlation between the ADC value and the cellular density has been verified. Briefly, the higher the cellular density is, the lower the ADC value will be in DWI, and vice versa [21]. For malignant tumors, they have a relatively high cellular density and therefore will produce a low ADC value in DWI, while for benign lesion, its density is generally low and thus will produce a high ADC value in DWI. Application of DWI in the diagnosis of breast lesions has been reported recently [14,16,22-24]. These studies showed that in malignant breast tumors, the ADC was significantly lower than that in benign tumors. These authors concluded that ADC might help to differentiate benign and malignant lesions with good specificity, and may increase the overall specificity of breast MRI, which is consistent with our results.

In ROC curves, the specificity of the ADC is dependent on the threshold value that determines the differentiation between benign and malignant tumors. In our study, we obtained two threshold values: 1.24 ± 0.25 × 10^-3^mm^2^/s (b = 500) with 93% sensitivity and 100% specificity; and 1.20 ± 0.25 × 10^-3^mm^2^/s (b = 1000) with 96% sensitivity and 97% specificity. The threshold of ADC value in our study was close to the data reported by Luo JD [16] (1.22 × 10^-3^mm^2^/s) and Rubesova E [14] (1.13 ± 0.10 × 10^-3^mm^2^/s). The reason of difference was that in our study we chose b = 500 and b = 1000 while in Luo JD's study, b = 0 and b = 800 and in Rubesova E's study, three-dimensional fast low-angle shot (3D-FLASH) with contrast injection was applied. An exception in our study is that one breast malignant phyllode had a high ADC value (b = 500, 1.72 ± 0.14 × 10^-3^mm^2^/s; b = 1000, 1.73 ± 0.14 × 10^-3^mm^2^/s), which was similar to that in Woodhams R's report (1.67 ± 0.59 × 10^-3^mm^2^/s) [12]. The reason of a high ADC in tumor was that in this case the lesion was mainly liposarcoma histologically and the liposarcoma, similar to adipose tissue, has a relatively high ADC value.

Another important aim in our study is try to study the DWI changes in tissues adjacent to breast carcinoma. It is well known that over the past 30 years in the field of breast carcinoma surgery, the extent of surgery has been progressively reduced, which leads to less disfigurement and a significant improvement in life quality of patients [25]. From an oncological perspective, outcome of conservative breast surgery was found to be equally effective when compared with mastectomy [26]. However, tumor recurrence after breast-conserving surgery still remains a problem [27]. Many studies [28-31] reported that obtaining negative surgical margins in conservative breast surgery influenced the incidence of local disease recurrence and probably overall survival. Preoperative evaluation of the change in peri-tumor tissue will plays an important role for the success of conservative operation.

Nowadays in clinical works, the preoperative estimation of the excision scope of breast tumor is generally made by surgeon according to his own experience, based on the results of mammography, ultrasound and conventional MRI. However, because the capacity for malignant growth is acquired by the stepwise accumulation of defects in specific genes regulating cell growth and tissue homeostasis [32], the genetic and molecular alteration prior to phenotypic changes in peri-tumor tissues usually could not be reflected by conventional mammography, ultrasound and MRI examinations. On the other hand, although intraoperative pathological diagnosis was regarded as an important standard second only to routine paraffin section for the evaluation of negative or positive margin of tumor, it can only be performed approximately 30 minutes post excision of the lesion and the decision must be made intraoperatively [33-37]. Routine paraffin section diagnosis is the "gold standard", however, the data will be decided after operation a couple of days. Therefore, it is essential to explore new techniques that should be noninvasive and could detect the change of peri-tumor tissue before operation. For these reasons, DWI, due to its distinct characteristics mentioned above, might provide some information on the microstructure change of breast tissues and probably becomes a potentially valuable method for evaluating the change of peri-tumor tissue in breast carcinoma.

In our present study we first applied DWI to compare the ADC value of malignant tumor with that of its peripheral tissue. We found that from the parenchyma of tumor to its peripheral tissue, which was layered with a thickness of 5 mm, the ADC values gradually increased and between the innermost layer 1 and other outer layers, there was a significant difference in their ADC values (*P *< 0.05), while there was no difference from layer 2 to layer 4. Further comparison study showed that only ADC value of layer 1 was significantly lower than that of normal contralateral breast tissue. Although from layer 2 outwards, there was no statistical difference among different layers, it can not be concluded that the tissue in layer 2, or even outer layers, was completely normal, since the tumor intrude into surrounding tissue with infiltrative growth pattern but we considered each layer as a homogeneous tissue while we performed statistical analysis.

Besides, since this work was basically a methodological study and our aim was to study the change of ADC values change in peri-tumor tissue by the novel MRI technique, all the lesions recruited in our study were from mastectomy and relatively large in size. Therefore, further researches are needed to assess whether the results and threshold in the present study could be also applied to the smaller lesions.

## Conclusion

In conclusion, our study showed that the ADC value was a sensitive and specific parameter that can help to differentiate benign and malignant breast lesions with a threshold value of 1.24 ± 0.25 ± 10^-3^mm^2^/s (b = 500) and 1.20 ± 0.25 ± 10^-3^mm^2^/s (b = 1000). Our results also showed that the ADC values increased gradually from the tumor to peri-tumor to normal tissues and there existed a region with a thickness of about 5 mm surrounding the border of tumor (by routine MRI), which has an abnormal ADC value, while beyond this region, the ADC value of tissue return to a normal range gradually.

Our next stage studies will include: 1) Measure ADC value of each ROI layer with a thinner thickness to detect the gradual change from carcinoma to normal tissue; 2) Compare the examination of DWI with that of pathological histology about the change of peri-tumor tissue in breast carcinoma and study the correlation of diagnosis between these two methods.

## Abbreviations

ADC: Apparent Diffusion Coefficient; DWI: Diffusion-Weighted Imaging; MRI: Magnetic Resonance Imaging; ROC: Receiver Operating Characteristic; ROI: Region Of Interest.

## Competing interests

The authors declare that they have no competing interests.

## Authors' contributions

YLZ conceived of the study concept and participated in its design, MRI imaging data acquisition, literature research, statistical analysis, manuscript drafting and editing, approval for important intellectual concepts. XYH carried out the MRI imaging data acquisition, participated in the literature research and manuscript drafting and editing. HWD participated in the clinical study, imaging data analysis and manuscript drafting and editing. YZ participated in the clinical study, imaging data analysis, literature research. XC participated in the clinical study, data acquisition, statistical analysis. PW participated in the clinical study, data acquisition, literature research and statistical analysis. YMG conceived the study concept and participated in design, manuscript drafting and editing data analysis and interpretation, approval for important intellectual concepts. All authors read and approved the final manuscript.

## Pre-publication history

The pre-publication history for this paper can be accessed here:

http://www.biomedcentral.com/1471-2407/9/18/prepub
